# Suppression of non-selected solutions as a possible brain mechanism for ambiguity resolution in the word fragment task completion task

**DOI:** 10.1038/s41598-022-05646-5

**Published:** 2022-02-03

**Authors:** Maxim Kireev, Alexander Korotkov, Ruslan Masharipov, Maya Zheltyakova, Denis Cherednichenko, Valeria Gershkovich, Nadezhda Moroshkina, Natalia Slioussar, Victor Allakhverdov, Tatiana Chernigovskaya

**Affiliations:** 1 Institute for Cognitive Studies, Saint Petersburg State University, Saint Petersburg, Russia; 2 N. P. Bechtereva Institute of the Human Brain of the Russian Academy of Sciences, Saint Petersburg, Russia; 3 National Research University “Higher School of Economics”, Moscow, Russia

**Keywords:** Cognitive control, Consciousness, Language, Problem solving

## Abstract

Brain systems dealing with multiple meanings of ambiguous stimuli are relatively well studied, while the processing of non-selected meanings is less investigated in the neurophysiological literature and provokes controversy between existing theories. It is debated whether these meanings are actively suppressed and, if yes, whether suppression characterizes any task that involves alternative solutions or only those tasks that emphasize semantic processing or the existence of alternatives. The current functional MRI event-related study used a modified version of the word fragment completion task to reveal brain mechanisms involved in implicit processing of the non-selected solutions of ambiguous fragments. The stimuli were pairs of fragmented adjectives and nouns. Noun fragments could have one or two solutions (resulting in two words with unrelated meanings). Adjective fragments had one solution and created contexts strongly suggesting one solution for ambiguous noun fragments. All fragmented nouns were presented twice during the experiment (with two different adjectives). We revealed that ambiguity resolution was associated with a reduced BOLD signal within several regions related to language processing, including the anterior hippocampi and amygdala and posterior lateral temporal cortex. Obtained findings were interpreted as resulting from brain activity inhibition, which underlies a hypothesized mechanism of suppression of non-selected solutions.

## Introduction

We live in a world where almost all visual images, linguistic expressions and other objects are inherently ambiguous or uncertain. For instance, successful language processing requires resolving ambiguities at every level, including phonological, orthographic, semantic and syntactic. Therefore, we are constantly engaged in the process of ambiguity resolution, and different aspects of this process are extensively studied in psychology, linguistics and other cognitive sciences.

One of the questions, which is still unresolved, concerns the role of suppression and the brain mechanisms underlying it that are involved in ambiguity resolution. Some authors assume that when one solution is selected, nothing in particular happens to the alternative non-selected ones (e.g.^1,2^). The other theories claim that they must be actively suppressed (e.g.^3–6^). For instance, semantic ambiguity is associated with slower recognition and response times, and this is traditionally explained by competition between multiple unrelated meanings for selection or retrieval^7,8^. According to the models like^3–6^, the disambiguation of the meaning of an ambiguous word could be realized via suppression of the non-selected meanings, which allows avoiding interference-related conflict in subsequent processing of the same ambiguous stimulus in the same context.

The brain activity associated with semantic ambiguity resolution is relatively well studied and involves greater activation of brain areas specialized for semantic processing and executive functions including posterior temporal and inferior frontal regions of the left brain hemisphere^8–14^. The activity in these regions increased when highly ambiguous sentences were compared with the ones low in ambiguity. Different authors considered the possible role of the LIFG in processing multiple meanings to be related to conflict monitoring between these meanings or to combinatorial processing associated with selecting contextually appropriate ones. The posterior temporal region activity was attributed to the reinterpretation process needed to disambiguate and to select the meaning most relevant for the current context. The greater involvement of these brain areas, as well as greater reaction times for processing such ambiguous stimuli, are usually regarded as a brain signature of the semantic ambiguity disadvantage.

However, in some cases, ambiguity can be a source of advantage rather than disadvantage^15^. For instance, in the lexical decision task, participants can categorize items as real words faster and more accurately if they are ambiguous words than if they are unambiguous words^16,17^. Presumably, in such tasks as lexical decision, solutions can be found relatively early in processing (e.g.^18^), and do not necessarily involve a semantic competition. This leaves an opportunity that the process of disambiguation (i.e. selecting one of the meanings) could occur without conscious awareness. Indeed, in the majority of cases the contextually appropriate meaning pops up in the reader’s or listener’s mind without a conscious recognition of other possible meanings. One of the possible underlying mechanisms was proposed by Allakhverdov^19^. According to his model, the cognitive system tries to select only one meaning for awareness. All other meanings are actively suppressed, making them less accessible, or “negatively chosen”.

Thus, initially multiple meanings are activated^20^, and then an automatic uncontrolled selection process^21,22^\is involved in choosing one meaning for awareness. The first question that we address in the current study is whether non-selected solutions are actively suppressed. As we show below, we do so using a modified version of the word fragment completion task to avoid various complications associated with the tasks dealing with semantic ambiguity and to focus on implicit processing. The second question concerns possible aftereffects of the positive choice for awareness and the negative choice: if suppression is involved, we expect not only positive aftereffects of the former, but also negative aftereffects of the latter. In particular, we will tend to reproduce the previously selected meaning and not to select the competitors (i.e. negatively chosen meanings).

To find out whether suppression is involved in automatic ambiguity resolution, it is useful to look at another phenomenon traditionally assumed to involve suppression: at retrieval-induced forgetting (RIF). The core assumption concerning RIF is that inhibitory processes are recruited during the control of memory selection and retrieval in order to overcome possible interference^23^. The typical RIF paradigm includes three phases. In the study phase, participants are asked to study word pairs that consist of a category name and a word that belongs to that category. Then participants go through retrieval-induced practice several times: for some items, participants are shown the category name and the first two letters of a studied word from that category and are asked to remember the studied word. In the test phase, participants are asked to remember all studied items. In general, unpracticed words from the same category are remembered less accurately than the baseline of unpracticed words from the different categories.

This effect was observed for various stimuli ranging from visual objects and actions to autobiographical memories. Johnson and Anderson^24^ also showed that if participants generated associates to the subordinate verb meaning of homonymous words several times (e.g., *prune t_ _m* for *prune trim*), they were less likely to use the dominant noun meaning in a free association test with novel test cues (e.g., *yogurt f__* for *fruit*, which is related to the noun *prune*). These results are very close to the studies of lexical ambiguity resolution showing that a contextually inappropriate meaning may be suppressed below the baseline^25^.

Inhibition may influence not only conceptual, but also perceptual representations. There is some evidence that lexical competition impairs retrieval in the word fragment completion task^26^. Perfect et al.^27^ demonstrated the forgetting effect only for the category generation task and the category verification task, whereas there was no effect for perceptual identification, category-cued perceptual identification, and word-stem completion tasks. However, data from experiments involving direct and indirect word fragment completion tasks suggest that perceptually driven tasks can show significant RIF effects, demonstrating suppression of perceptual traces and proving that lexical representations are involved when participants complete word fragments^28^.

Moreover, Healey et al.^29^ demonstrated a RIF effect using the word fragment completion task with orthographically similar words. They showed that suppression is necessary whenever competition is involved and that suppression of competing information can occur even after a single retrieval episode. In other words, a single presentation of a word fragment which has several solutions could induce suppression of non-selected solutions. We relied on this finding in the current study and confirmed it using a different experimental design.

Given the focus of the current study on processing of non-selected competitors in case of ambiguous information, the word fragment completion task appears to be optimal because it allows overcoming inherent limitations of the tasks relying on semantic ambiguity. Semantic ambiguity would simultaneously involve a number of additional processes like interference between multiple meanings, meaning selection, and greater executive efforts needed to overcome or resolve the conflict between multiple meanings. All these processes presumably would attenuate the possible suppression of non-selected meanings, which would be impossible to disentangle from the abovementioned processes.

Therefore, the aim of the current study was to investigate brain mechanisms involved in the processing of non-selected solutions. We used fragmented word pairs: adjectives and nouns, in which nouns could have one or two solutions and adjectives created contexts biasing one solution. We hypothesized that this task would induce automatic selection of the contextually appropriate variant of completion and suppression of the alternative solution. We expected that if a suppression mechanism is involved in processing of non-selected solutions, we would observe inhibitory effects in the functional brain activity revealed by functional MRI. We were also going to look for possible aftereffects of the negative choice during the second presentation of ambiguous word fragment, both in behavioral and in neuroimaging data.

## Materials and methods

### Participants

Forty eight volunteers (20 males, 28 females) with the mean age of 26.7 ± 5.3 took part in the study. All participants were right-handed native Russian speakers with no history of psychological or neurological diseases. Their handedness was assessed with the Edinburgh Handedness Inventory^30^. The study and all methods were carried out in accordance with the relevant ethical guidelines and regulations. All volunteers gave a written informed consent before the beginning of the study. The Ethics Committee of the N.P. Bechtereva Institute of the Human Brain (Saint Petersburg, Russia) approved all procedures.

### Stimuli and procedure

The experimental task was to complete Russian adjective-noun fragments with missing letters. For example, the fragments like *s-hoe vi-o* or *s-hoe -ino* were shown on the screen, and participants had to identify the missing letters by saying the complete phrase aloud: *suhoe vino* ‘dry wine’. To induce implicit processing of stimuli and to avoid conscious awareness of possible multiple solutions, subjects were instructed to produce aloud the first solution came to their mind. For adjectives, there was always only one completion option. For nouns, there were one or two, depending on the experimental condition, but the adjective strongly favored one of them in each fragment. In the examples above, *vi-o* can be completed only as *vino* ‘wine’ (control condition), while *-ino* can also be completed as *kino* ‘movie’ (ambiguous condition).

Adjective-noun phrases used as stimuli were created in the following way. At first, using the StimulStat database^31^, we selected pairs of nouns that differed only by one letter, as in *vino* ‘wine’ and *kino* ‘movie’. All nouns were 4–5 letters long, their frequency ranged from 5 to 100 ipm (according to the frequency dictionary^32^). In every pair, the difference in frequency between the two nouns was never greater than 50 ipm. For every noun, we selected an adjective that frequently cooccurs with it and is incompatible with the other noun in the pair. For example, *suhoe* ‘dry’ was selected for the noun *vino* ‘wine’, and *interesnoe* ‘interesting’ for *kino* ‘movie’.

To check that the selected adjectives are indeed strongly associated with one noun in the pair, but not with the other, we conducted a pilot study with 22 volunteers (adult healthy speakers of Russian who provided an informed consent). They did not take part in the main experiment. In this study, adjective-noun fragments in the ambiguous condition were shown on the computer screen one by one in a random order. Participants were asked to complete them as fast as possible, though we did not include time limits in the experimental procedure. As a result, we selected 48 stimulus sets (consisting of two nouns and two adjectives) relying on the following inclusion criteria: more than 70% of correct completions for both adjective-noun combinations within the 5 s time interval after the fragment presentation.

Out of these 48 sets, 15 included noun pairs differing by the first letter. In seven sets, the last letters of the nouns were different; in the remaining 26 sets, the difference was in the middle letter. For every set, we created four stimulus fragments, two in the ambiguous condition (e.g. *s-hoe -ino, i-teresnoe -ino*) and two in the control condition (e.g. *s-hoe vi-o, i-teresnoe ki-o*). In the two ambiguous fragments, the noun stimuli were identical (e.g. *-ino*). So, if one of them is presented after the other, we can study how one and the same ambiguous stimulus is processed for the first and for the second time in two different contexts (created by adjectives).

In the fMRI study, every participant was asked to complete 96 fragments, i.e. all adjective-noun combinations we created (in one of the two experimental conditions). Two combinations from the same set were always separated by three other trials and were shown in the same condition. I.e. those participants who saw *s-hoe vi-o* also saw *i-teresnoe ki-o* (both fragments in the control condition), those who were presented with *s-hoe -ino* were also presented with *i-teresnoe -ino* (both fragments in the ambiguous condition)*.* This gives us four options for every stimulus set: e.g. presenting the ‘wine’ fragment before or after the ‘movie’ fragment in the ambiguous or in the control condition. Using the Latin square principle, we created four experimental lists. For every list, we generated three different stimulus presentation sequences making sure that two fragments from every set are separated by three trials.

**Figure 1:**
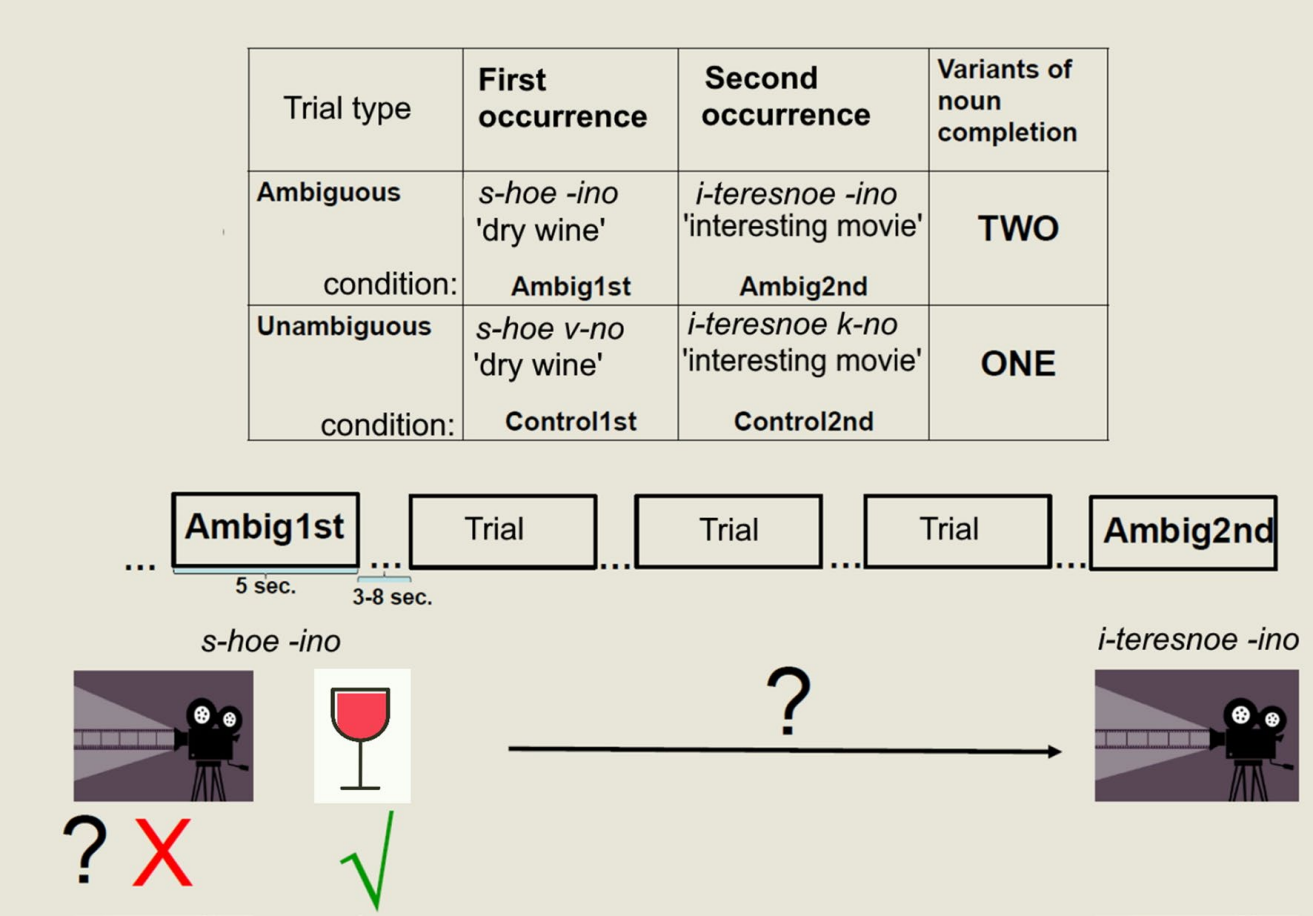
The experimental design of the fMRI study. The table illustrates four different trial types. The pictures in the lower part illustrate two variants of completion of the noun *-ino* (*vino* ‘wine’ or *kino* ‘movie’).

As a result, we had four types of experimental trials (see Fig. 1): (1) *Ambig1st*—the first fragment from the set in the ambiguous condition (for example, *s-hoe -ino*); (2) *Ambig2nd*—the second fragment from the set in the ambiguous condition (for example, *i-teresnoe -ino*); (3) *Control1st*—the first fragment from the set in the control condition (e.g. *s-hoe vi-o*); (4) *Control2nd*—the second fragment from the set in the control condition (e.g. *i-teresnoe ki-o*). In the *Ambig1st* trials, the noun can be completed in two different ways, but the context (i.e. the adjective) facilitates the unconscious choice of one variant (e.g. the word *vino* ‘wine’ in Fig. 1). We aimed to test whether the second variant (the word *kino*) is suppressed as a result of this choice. To do so, we compared *Ambig1st* trials to *Control1st* trials in which no suppression could be expected (but the same adjectives and nouns were used to avoid any confounding factors). We also wanted to test if the selection process in the *Ambig1st* trial complicates the selection of the supposedly suppressed variant in the following *Ambig2nd* trial. To do so, *Ambig2nd* trials were compared to *Control2nd* ones in which no effects of earlier suppression could be expected. The interval of three trials was selected as relatively short, but not instantaneous.

Each adjective-noun fragment was presented on the screen for 5 s. During this time, a participant had to press the MR-compatible controller button and to say aloud the completed phrase. Button pressing was introduced to the task in order to measure possible changes in reaction times, since we could expect negative aftereffects in the *Ambig2nd* condition caused by hypothesized suppression of non-selected completion in the *Ambig1st* condition. Oral answers were recorded and subsequently analyzed to identify errors in finding solutions for word fragments. We expected that memory blocking effects would be associated with intrusion errors, i.e. repeating the solution found for the fragmented noun in the *Ambig1st* condition, but irrelevant for the *Ambig2nd* condition.

The stimulus did not disappear after the button was pressed, always remaining on the screen for 5 s. During interstimulus intervals, a fixation cross was presented on the screen. To improve the sample rate of the hemodynamic response function (HRF) their length randomly varied between 3 and 8 s with the 500 ms equiprobable step. The total duration of one fMRI session was 16.8 min.

After the experiment was over, participants received a printed list of all stimuli (in the presentation order) and were asked to indicate for which ambiguous fragments they realized that two completion options were possible. Participants reported a very small number of such cases, which were excluded from the subsequent analysis. Of course, we could not guarantee that they remembered all such cases accurately, but wanted to do as much as we could to exclude them, since we were specifically interested in the unconscious processes of ambiguity resolution in this study.

### fMRI Image acquisition procedure and image processing

All structural and functional images were acquired using a 3T Phillips Achieva scanner with the 8-channel receiving coil (Philips Medical Systems, Best, Netherlands). Stimuli were presented using the specialized system for presenting visual stimuli NordicNeurolab, which allows synchronizing stimulus presentation (using E-prime) with participants’ responses (button presses) and BOLD signal registration in the scanner. Oral responses were recorded via an MR-compatible microphone with a noise reduction system.

To obtain structural images of each participant’s brain T1-weighted pulse sequences of high resolution were used (T1W-3D-FFE; [TR] = 2.5 ms; [TE] = 3.1 ms; flip angle = 30°; 130 slices, [FOV] = 240 × 240 mm; matrix = 256 × 256, slice thickness = 0.94 mm). Functional images of BOLD signal changes were registered with echo-planar imaging (EPI) sequences (so-called dynamic scans): the registration time of 32 axial slices was 2 s (TE = 35 ms) with the FOV 208 × 208, flip angle = 90° and pixel size 3 × 3 mm. The thickness of the slices was equal to 3 mm. Structural MRI images were used in the analysis of fMRI-data in two cases: (1) for spatial normalization of individual data into a standard stereotactic space; (2) for creating a mean grey matter image used as an “explicit mask” in the fMRI-data analysis—this allowed performing the statistical analysis only for those voxels, which were localized in the grey matter for all participants.

Prior to the statistical analysis individual dynamic scans were preprocessed in the following stages: (1) realignment of all images relative to the first dynamic scan with the calculation of head movement parameters; (2) slice-time correction; (3) normalization of functional images into a standard stereotactic space with the coregistration of structural T1-image with the first dynamic scan and its segmentation; (4) Gaussian smoothing (8 mm, FWHM). Preprocessing and statistical analysis were performed using the SPM12 software package (Statistical parametric mapping 12) running in Matlab (2012b, Mathworks Inc., Natick, MA, USA).

### Statistical analysis of fMRI data

On the first level of the statistical analysis, the brain activity associated with the experimental task was estimated individually for each participant. The generalized linear model (GLM)^33^ included regressors corresponding to the four experimental conditions: *Ambig1st, Ambig2nd*, *Control1st* and *Control2nd*. Trials in which participants gave incorrect responses or did not respond at all constituted the regressor «Mistake». Ambiguous trials for which participants subsequently reported recognizing two completion options were also removed from the analysis by modeling a separate regressor («DoubleMeaning»). On average, 4 (SD 3.5) out of 48 ambiguous fragments were identified as «DoubleMeaning» regressors. Additionally, to account for head movements, the GLM included six regressors representing movement parameters, calculated on the realignment stage of preprocessing.

The standard hemodynamic response function and stimulus onset times were used to calculate regressors. Beta-coefficients, obtained for linear contrasts between compared experimental conditions on the individual level, were used in the second (group) level of the statistical analysis. They represented BOLD signal changes in relative units. The group analysis was performed in a voxel-wise manner using the GLM model for the random effect analysis.

To test the hypothesis concerning the involvement of neural mechanisms responsible for processing non-selected solutions of ambiguous word fragments, two contrasts of experimental conditions, scaled to a percent signal change relative to the whole-brain BOLD signal^34^, were analyzed: *Ambig1st* > *Control1st* and *Ambig1st* < *Control1st*. The linear contrasts between *Ambig2nd* and *Control2nd* were analyzed to test the assumption regarding the proactive interference caused by recent solutions found to the same fragmented nouns, which is irrelevant to the current context induced by the new fragmented adjective.

At the group level of analysis, a Parametric empirical Bayes with the ‘global shrinkage’ prior (as implemented in the SPM12) was applied^35^. The ‘global shrinkage’ prior represents a prior knowledge that on average over all voxels, there is no global experimental effect^36^. The effect refers to the percent signal change difference between conditions (i.e. the contrasts obtained during the first-level analysis). In accordance with Bayesian statistics, the conclusion about the presence or absence of the effect of interest in a group of participants is based on the calculation of the posterior probability of the effect given the obtained data. If the posterior probability of finding a non-zero effect is high, *P*(*Effect* > 0|*Data*) > *P*_*threshold*_, then one can ‘accept’ the alternative hypothesis. In contrast, using classical frequentist inference, one can only ‘reject’ the null hypothesis.

To improve the visualisation of voxels that have posterior probabilities close to one, the posterior probability maps were converted to the Log Posterior Odds maps, log*P/*(1 − *P*)^37^. The posterior probability threshold was *P*(*Effect* > 0|*Data*) > 0.99, which corresponds to Log Posterior Odds > 5. The advantage of the Bayesian approach we used is that it automatically accounts for multiple comparisons, shrinking extreme values (which are unlikely a priori) to zero. Thresholding posterior probability maps corresponds to the False Discovery Rate correction in the frequentist framework^35^.

## Results

### Behavioral data

Analysis of reaction times (RT) using non-parametric Mann–Whitney U Test didn’t reveal significant changes between *Ambig1st* and *Control1st* trials (group medians: 1777 ms and 1730 ms, *p* = 0.75), between *Ambig2nd and Control2nd *(group medians: 1907 and 1665 ms, p = 0.053), as well as between *Ambig1st* and *Ambig2nd* trials (*p* = 0.22).

The number of “Double meaning” trails in which subjects reported that they noticed that presented fragmented noun had two solutions didn’t significantly differed between *Ambig1st* and *Ambig2nd* conditions (group means percentage from the total number of trials for *Ambig1st and Ambig2nd*: 4.2% and 3.8%, *p* = 0.53).

Analyzing completion errors, we divided them into: (1) omissions, i.e. not giving any response in the allotted time (both for the first and the second occurrence of the fragmented noun); (2) intrusions, i.e. repeating the previous solution which is inappropriate in a new context; (3) wrong solutions, i.e. solutions not associated with the previously presented and orthographically similar fragmented noun. Wrong solutions were significantly more numerous in the *Ambig1st* condition (group mean 7.7%, standard deviation (SD) 6.7%) than in the *Control1st* condition (group mean 1.9%, SD 3.3%), as assessed by Mann–Whitney U test (z = 4.93, *p* < 0.001). The analogous analysis of omission errors revealed significantly greater (z = 2.5, *p* = 0.01) error rates in the *Ambig1st* condition (group mean 7.7%, SD 6.8%) compared to the *Control1st* (group mean 4.5%, SD 5.4%).

Intrusion errors in the *Ambig2nd* condition, associated with replicating the solution from the *Ambig1st* condition, were observed for 36 subjects, while the other 12 subjects did not demonstrate intrusion errors at all. The group mean percentage of intrusion errors was 7.7% with SD 7.9%. Intrusion can be expected only in the Ambig2nd condition, so, instead of comparing its incidence in different conditions, we compare intrusion errors to other wrong solutions below. The wrong solutions in the *Ambig2nd* and *Control2nd* conditions did not differ significantly and were relatively infrequent, with the group means of 1.1% (SD 2.1%) and 1.6% (SD 3.3%) correspondingly. Omission errors in the *Ambig2nd* condition (group mean 8.1%, SD 8.3%) were significantly more numerous (z = 2.4, *p* = 0.02) in comparison with the *Control2nd* condition (group mean 4.5%, SD 6.6%). The incidence of omission and intrusion errors did not differ significantly in the *Ambig2nd* condition, but both types were more widespread than wrong solutions (z = 5.38, *p* < 0.001 and z = 5.21, *p* < 0.001, respectively). Finally, there was no significant difference between the *Ambig1st* and *Ambig2nd* in terms of omission errors.

### Neuroimaging results

**Figure 2:**
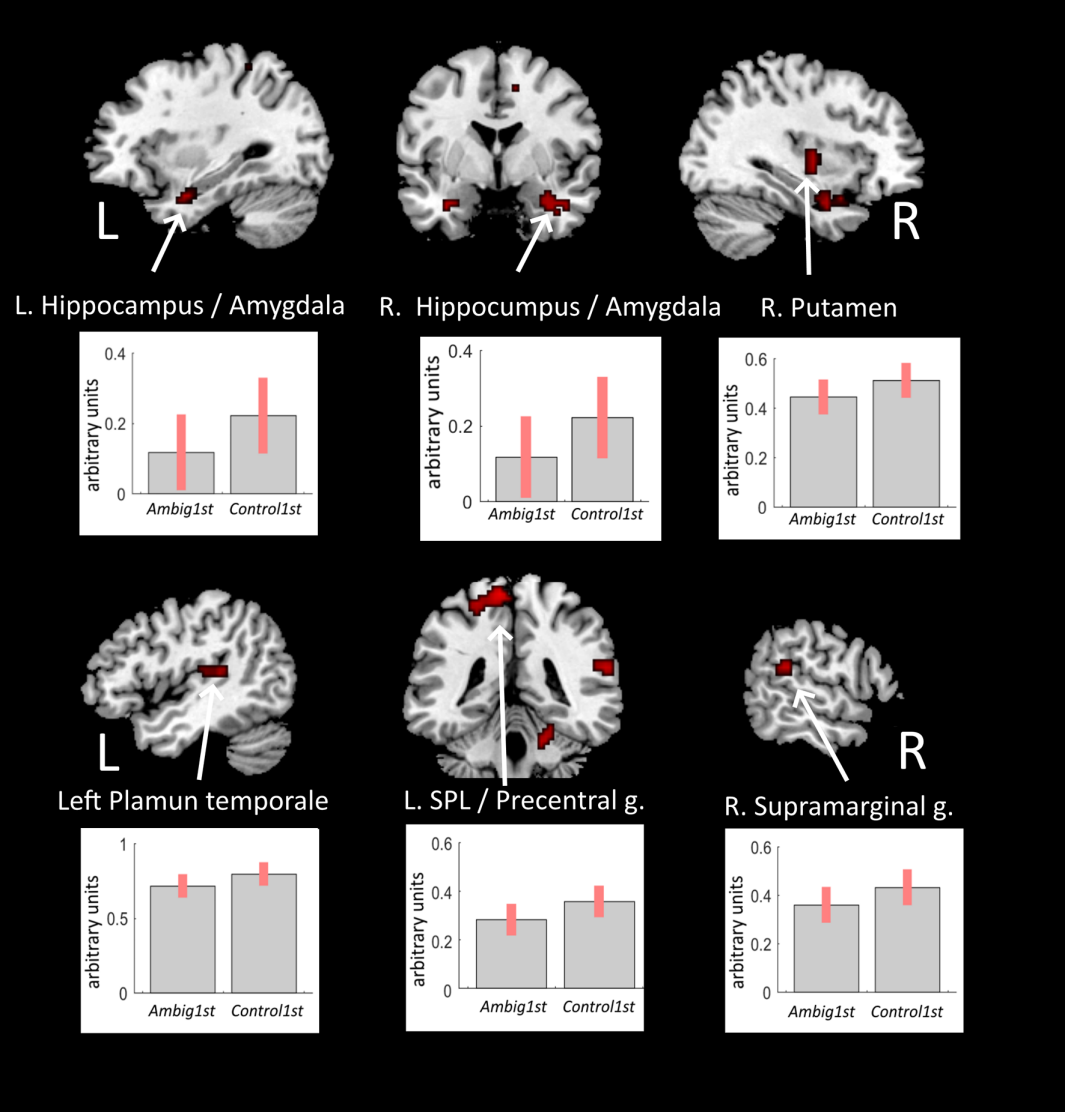
Changes in the BOLD signal associated with processing of non-selected solutions for the word pair fragments. Clusters of significant t-contrast *Control1st* > *Ambig1st* presented over a template brain image.

Significant BOLD signal changes were revealed in the *Control1st* > *Ambig1st* linear contrast using Bayesian inference. Finding solutions for fragmented word pairs in which nouns had two possible completions was characterized by reduced BOLD signal within the anterior hippocampus and amygdala, the planum temporale bilaterally, as well as in the right supplementary motor area, the left superior parietal lobule and precentral gyrus, the left precuneous, right cuneous and cerebellum. (Fig. 2, Table 1). The reversed *Ambig1st* > *Control1st* linear contrast did not reveal any significant changes in the BOLD signal.

**Table 1:** Decreases in the BOLD signal associated with processing of a non-selected solution revealed in the *Control1st* > *Ambig1st* contrast.

Brain region	k	Log odds	Peak MNI coordinates		
			x	y	z
**Contol1st > Ambig1st**					
Left hippocampus/amygdala	14	6.6	− 30	− 4	− 25
Right hippocampus/amygdala/ITG	51	6.9	39	8	− 25
Right SMA/Precentral gyrus	31	7	12	− 13	50
Right Putamen/Insula	18	6.3	+ 33	− 10	2
Right planum temporale/supramarginal gyrus	28	7.2	60	− 37	20
Left SPL/precentral gyrus	189	8.8	− 24	− 43	62
Left planum temporale/Heschl’s gyrus/parietal opeculum	24	5.9	− 42	− 25	14
Left precuneous	28	6.25	− 17	− 73	17
Right cuneal cortex	39	5.9	18	− 73	23
Right cerebellum	18	6.3	18	− 40	− 28

Denotations: *L/R* left/right hemisphere, *k* cluster size in voxels, *ITG* inferior temporal gyrus, *SMA* supplementary motor area, *SPL* superior parietal lobule.

**Figure 3:**
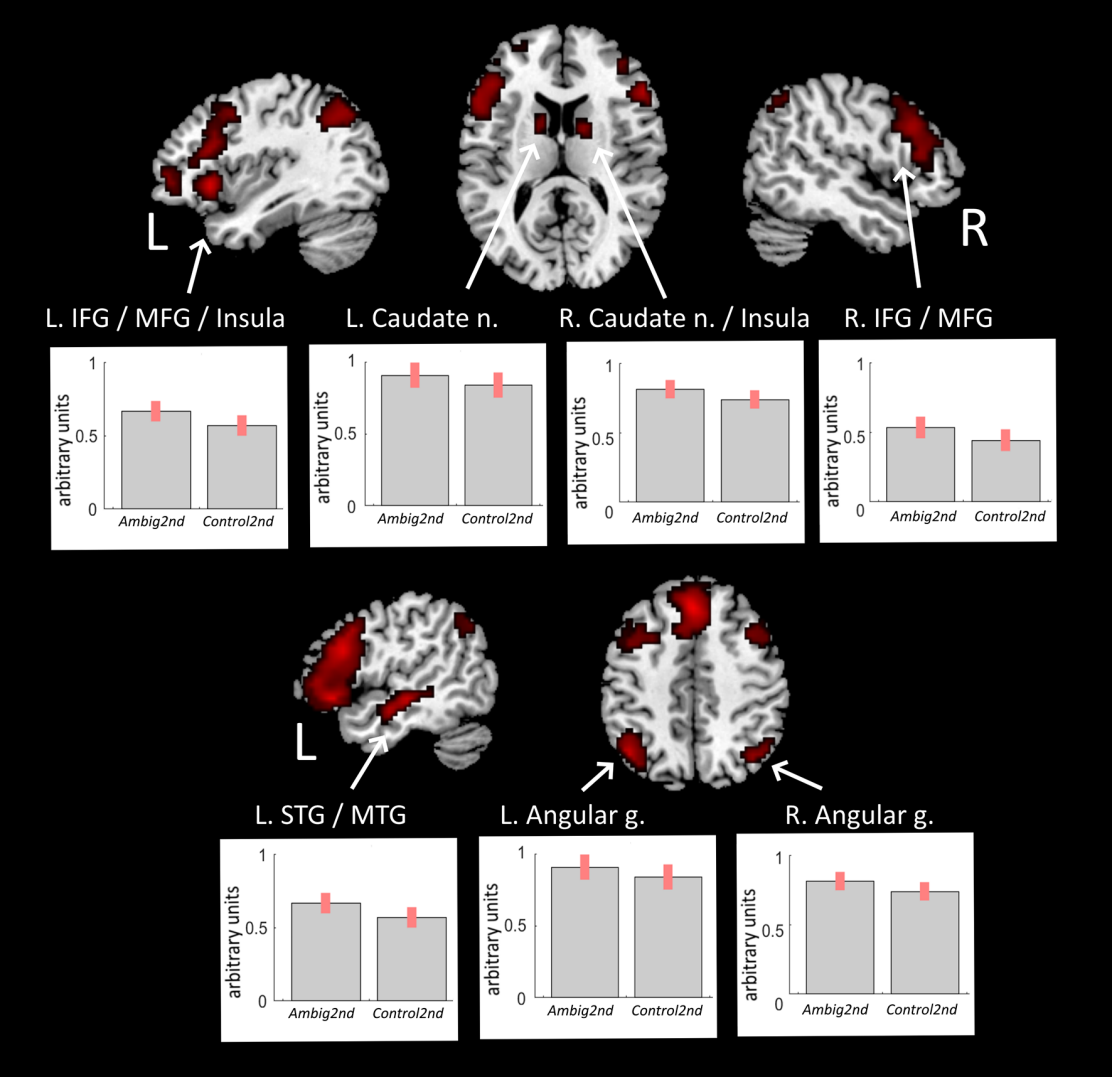
Enhanced BOLD signal associated with finding solution in Ambig2nd condition when fragmented noun having two solutions was paired with new fragmented adjective making previous solution to this nous inappropriate. Denotations: *L/R* left/right hemisphere, *IFG* inferior frontal gyrus, *MFG* middle frontal gyrus, *STG* superior temporal gyrus, *MTG* middle temporal gyrus, *g*. gyrus, *n*. nucleus.

Finding a solution for the second occurrence of a fragmented noun paired with a new fragmented adjective in the *Ambig2nd* condition, as compared with the *Control2nd* condition, revealed enhanced BOLD response in the fronto-parietal, temporal and basal ganglia regions including: the inferior frontal gyrus (IFG), the middle frontal gyrus (MFG), insular cortex and caudate nucleus bilaterally, as well as the left superior and middle frontal gyri, left precuneus and thalamus (Fig. 3, Table 2). The reversed *Ambig2nd* > *Control2nd* linear contrast did not reveal any significant changes in the BOLD signal.

**Table 2:** Increases of the BOLD signal associated with proactive interference between the previously chosen solution inappropriate in the new context and the correct alternative solution for fragmented word pairs in the *Ambig2nd* condition.

Brain region	k	Log odds	Peak MNI coordinates		
			x	y	z
**Ambig2nd > Control2nd**					
L. IFG/MFG/Insula/ (BA 44/45)	897	13.4	− 51	26	− 7
L SFG/Paracingulate gyrus	424	14.4	0	32	47
R. IFG/MFG (BA 44/45)	289	10	54	29	14
R. Frontal pole	13	5.9	39	44	14
R. Insula/Caudate nucleus	104	9.6	36	23	2
L. Caudate nucleus	38	6.7	− 12	8	8
L STG/MTG (BA 21)	109	12.3	− 51	− 22	− 7
Posterior cingulate gyrus	11	7.6	− 3	− 10	29
L. Thalamus	13	6.5	− 3	− 28	5
L. Angular gyrus/SPL/Lateral occipital cortex	157	11.4	− 42	− 61	44
R. Angular gyrus/Lateral occipital cortex	64	8.6	36	− 67	50
L. Precuneous/SPL	13	5.9	− 6	− 70	35

Denotations: *L/R* left/right hemisphere, *k* cluster size in voxels, *BA* Brodmann area, *IFG* inferior frontal gyrus, *MFG* middle frontal gyrus, *STG* superior temporal gyrus, *MTG* middle temporal gyrus, *SPL* superior parietal lobule.

## Discussion

The main finding of the current study is that completion of fragmented nouns that had two solutions is characterized by bilaterally reduced BOLD signal within brain areas responsible for language processing, including the anterior hippocampus, supramarginal gyrus and the planum temporale. In accordance with recent meta-analytic studies^38^, the anterior hippocampus is responsible for encoding and retrieval during word processing: it underlies the process of binding new information with prior knowledge. It is also associated with retrieval of concepts from semantic memory. Therefore, we consider the observed reduction of its activity as a reflection of suppressing an inappropriate solution for a word fragment.

In this respect, a distinction between identification and production tasks^39^ should be noted. In identification tasks (perceptual identification, lexical decision etc.), participants attempt to identify the cue or some of its features. They involve search processes that eventually lead to one particular representation in the memory. Production tasks (e.g. word-stem completion) presuppose competition of several candidates for the response and a selection mechanism to choose a single one. The recognition of a printed word results from the match between a letter string and a lexical representation. This match allows the reader to access the mental lexicon^40^. If a fragment of a printed word can be matched only with one entry in mental lexicon, participants would complete it based on one representation, and this would be an identification task. If multiple solutions are possible, this would become a production task^41^. The word fragment cue would evoke several lexical entries, which would call for a selection process.

If completion task supposes production for word-fragment with multiple solutions, it would result in competition between possible solutions for response, as well as selection of context appropriate variant for completion and suppression of non-selected solutions. Therefore, we interpret observed reduction of neuronal activity in brain areas responsible for word processing and production as an evidence towards the involvement of a suppression mechanism. These results also correspond to the reported reduction of hippocampal activity during effortful retrieval and memory search: a task-induced suppression of its anterior division was observed in several studies^42,43^. Intriguingly, the reduction of neuronal activity in the current study was also observed in the posterior temporal lateral cortex (including planum temporale) responsible for activating lexical–semantic information in the process of retrieving meanings from long term memory, i.e. word comprehension^44,45^. Taken together, these findings could be considered as evidence towards the suppression of non-selected solutions for ambiguous fragmented nouns when only one solution must be selected for oral production.

Obtained results also support the idea that inhibiting the hippocampus is a critical neurobiological mechanism responsible for preventing competing or interfering memories from intruding to awareness. Such purging allows not only to select and fixate one meaning of an ambiguous stimulus, but also to avoid proactively the interference from competing memories in the forthcoming behavior. In this sense, the observed reduction of the hippocampal activity is similar to comparable effects of retrieval-induced forgetting.

Moreover, a similar effect on brain activity was observed when some memories were effectively forgotten due to voluntary efforts to suppress them in the Think/NoThink paradigm^46^. Such suppression-induced forgetting of unwanted memories is positively associated with the downregulation of the hippocampal activity caused by the right dorsolateral prefrontal cortex (DLPFC), as demonstrated by several effective connectivity studies^47,48^. This downregulation can be exerted over the hippocampus or the motor cortex depending on the behavioral goal to suppress unwanted memories or actions, respectively^49^. All these findings point towards a critical role of the inhibitory control in controlling the content of awareness. For instance, the individual level of the hippocampal GABA concentration assessed by the MR spectroscopy was positively correlated with the degree of functional decoupling between the DLFPC and the hippocampus associated with effective forgetting^50^. Therefore, despite the apparent psychological differences between the willful forgetting and the automatic suppression of context-irrelevant solutions during ambiguity resolution, they may rely on very close neurophysiological mechanisms of inhibitory control. Although the nature of inhibitory control involvement in suppressing non-selected solutions of ambiguous stimuli should be further investigated in future research, the results of the current definitely study extend the view of the role played by inhibition in the automatic control of awareness.

The results of assessing the aftereffects of proactive interference at the second presentation of an ambiguous noun fragment partly corroborated the hypothesis that non-selected solutions suffer from suppression. When seeing an ambiguous fragmented noun for the second time, participants needed to use the previously non-selected solution for this stimulus. This led to the BOLD signal changes resembling the negative priming effect^29,51^ observed as an increase of activity levels in the DLPFC, the angular gyrus, the superior and middle temporal cortex, and the basal ganglia.

Revealed clusters of increased BOLD signal located within the inferior frontal gyrus and the middle temporal gyrus are usually associated with the fronto-temporal regions involved in the competition resolution between alternative meanings that is needed to disambiguate semantically ambiguous information^9–13^. Although there were no significant differences in reaction times between different conditions, intrusion errors (repeating previously found solutions) point to the presence of interference between possible solutions. Therefore, the revealed involvement of the frontal, parietal and temporal brain regions possibly reflects the conflict between the dominant, but contextually irrelevant solution and the previously suppressed solution that became relevant in the new context. Accessing a previously suppressed solution is effortful since one needs to overcome the initial decision not to choose this solution, i.e., to negatively select it^19^. Such disambiguation process could be associated with increased activity in caudate nuclei: their role in semantic disambiguation was previously demonstrated by Ketteler et al.^52^. Moreover, this disambiguation process might be executed via selective inhibitory control of response interference supported by caudate nuclei.

However, the revealed BOLD-based negative priming effect^29^ could also be explained as a negative aftereffect of positive selection. For instance, according to a version of the episodic retrieval theory^53^, processing operations tend to be repeated if they were applied to a particular stimulus in the past. Likewise, another model, the theory of event coding^29,54^, predicts that the second presentation of the same noun with a missing letter will induce an automatic retrieval of the response given to the previous presentation of such noun. This prediction was corroborated by an independent behavioral study using the same experimental design^55^. This study demonstrated that when subjects completed ambiguous noun fragments for the second time, they often repeated a previously chosen variant inappropriate in the new context. In this sense, revealed changes could be associated with greater efforts needed to select between two possible alternatives like it happens in free choice deceptive paradigms, demonstrating similar patterns of BOLD signal changes^56^. Therefore, the present study cannot fully disentangle possible effects of suppression of a non-selected solution vs. a reinstatement of a previously chosen response to a particular stimulus in the observed negative aftereffect. We also cannot exclude the possibility that both processes are simultaneously involved in producing these negative priming or semantic ambiguity-like effects, which should be addressed in future research.

## Concluding remarks

The revealed reduction in the BOLD signal within the hippocampi and posterior lateral temporal cortex associated with finding solutions for ambiguous fragmented words supported the hypothesis that non-selected solutions are suppressed. The reduction in hippocampal neuronal activity associated with suppressing retrieval of non-selected meanings is similar to episodic memory effects observed in the settings of retrieval induced forgetting. This allows to consider such induced suppression as a key mechanism for purging unwanted memories from awareness. We hypothesize that this may be a general neurophysiological basis for automatic inhibitory awareness control with inhibitory downregulation of the hippocampus underlying it. This substantially extends the current view on the role of the inhibitory control in the automatic processing of ambiguous information. Observed increased levels of local activity within the frontoparietal brain network, the temporal cortex and basal ganglia may be associated with the negative aftereffect of ambiguity resolution and could be caused both by the non-selected response suppression and by the automatic retrieval of the previous response to the repeated ambiguous stimulus, or by the combination of these two processes.
